# Non-imported malaria in non-endemic countries: a review of cases in Spain

**DOI:** 10.1186/s12936-017-1915-8

**Published:** 2017-06-29

**Authors:** Emilia Velasco, Diana Gomez-Barroso, Carmen Varela, Oliva Diaz, Rosa Cano

**Affiliations:** 1 National Center for Epidemiology, Carlos III Institute of Health, Madrid, Spain; 2 Consortium for Biomedical Research in Epidemiology and Public Health (CIBERESP), Madrid, Spain

**Keywords:** Malaria, Transmission mechanisms, Non-endemic areas

## Abstract

Spain declared the elimination of malaria in 1964. In non-endemic areas, the overwhelming majority of malaria cases are acquired abroad, and locally acquired infections are rare events. In Spain, malaria is a statutorily notifiable disease. During these fifty years more than ten thousand malaria cases have been reported, and about 0.8% of them did not have a history of recent travel. In this report, it was carried out a review of the ways in which malaria can be transmitted in non-endemic areas and a short description of the Spanish cases, aggregated by their transmission mechanisms. Four cases contracted malaria by mosquito bites; there were two autochthonous cases and two of “airport malaria”. The other 28 cases were: congenital malaria cases, transfusion-transmitted malaria, post-transplant cases, nosocomial transmission and cases in intravenous drug users. In addition, in 1971 there was an outbreak of 54 cases due to exposure to blood or blood products. So, while malaria usually is an imported disease in non-endemic areas, it should not be excluded in the differential diagnosis of persons who have fever of unknown origin, regardless of their travel history.

## Background

Malaria is endemic in 97 countries and it is the most prevalent vector transmitted disease worldwide. According to World Health Organization (WHO) estimates, the global tally of malaria in 2015 was 212 million new cases [1]. In non-endemic areas, the majority of cases are imported; however, occasionally, cases are reported in which there is no history of travel—which necessitates a search for other modes of transmission.

A number of different possibilities for infection exist. One example is by competent vectors, either imported (“airport malaria” or “baggage malaria”) or local (introduced malaria). The bite of a mosquito that has inadvertently been brought on board an airplane can lead to cases in the same airport or, if climatic conditions permit, in the surrounding area [2]. On occasion, the mosquito is able to survive inside the luggage, causing infections even at a considerable distance from the airport [3] (baggage malaria). These cases generally present during the summer, a period more favourable to the survival of the mosquito, and during which there is an increase in international air traffic. When the bite is from a local vector, the disease is known as “introduced malaria” [4], a term which makes reference to the first generation of cases contracted locally, but which have a strong epidemiological link to an imported case.

Other situations exist where transmission is not caused by a mosquito bite but rather by contact with the blood or tissue of an infected person, either naturally (congenital malaria) or due to a blood transfusion or another form of parenteral contagion (induced malaria [4]).

Congenital malaria can be contracted by the transfer of *Plasmodium* through the placental barrier during pregnancy, even if the infection of the mother was asymptomatic, or by exposure to infected maternal blood during delivery. During the first few days the newborn’s haemoglobin (which impedes the development of the parasites) and the maternal IgG antibodies have a protective effect, as such symptoms do not usually appear until between 10 and 20 days after birth. Occasionally, the infection resolves itself spontaneously [5]. In non-endemic countries, congenital malaria usually affects the children of expatriates [6].

For what is referred to as “induced malaria”, the reported cases in non-endemic countries have been primarily related to blood transfusions [7]. The occurrence of such cases is now very low as a result of donor screening. Cases of secondary malaria from organ transplant have been reported [8] at a lower frequency (the majority following kidney transplants with *Plasmodium falciparum* as the main agent involved [9]) as well as various forms of parenteral needle-related infection (injections with contaminated needles [10], failure to change needles for multi-dose medicines [11]) or through contact with contaminated objects (catheters [12], gloves [13], etc.).

## Cases in Spain

In Spain, malaria was historically an endemic disease with several thousand cases annually [14]. Halfway through the twentieth century, sanitary and socioeconomic improvements and the efforts made to control the vectors succeeded in interrupting the transmission of the disease. Malaria was declared officially eliminated in 1964 [15].

Since then, malaria has continued to be a disease which it is obligatory to report to the Spanish Epidemiological Surveillance Network. This data is managed by the National Centre for Epidemiology in the Carlos III Health Institute. During the years since elimination, more than 10,000 cases of malaria have been reported, [16] making it the disease most frequently imported into Spain. However, although they represent exceptional cases, there have been cases in which a history of travel to an endemic zone was absent.

### Introduced malaria

The last case of autochthonous malaria occurred in Spain in 1961 [17]. Since then there have been two reported cases of introduced malaria. In neither case was there a history of travel to areas of risk, nor did the patients live in close proximity to international airports. Other possible methods of infection were ruled out (history of surgery, invasive procedures or blood transfusions).

The first case appeared in September 2010 [18]. It concerned a 48-year-old woman who presented with fever. This was initially diagnosed as acute tonsillitis and was treated with antibiotics. Ten days later the patient’s condition worsened and she was hospitalized. Microscopic examination of a blood sample identified *Plasmodium * sp. parasites, and molecular testing identified them as *P. vivax.* The patient lived in an area where *Anopheles atroparvus* is present, although in that area there had been no reported cases of imported malaria in the preceding years. Despite an exhaustive investigation, it was not possible to identify the source of the infection.

The second case was in August 2014 when a 62-year-old man with high fever and muscular pains was hospitalized [19]. A microscopic examination revealed the presence of intraerythrocytic parasites and a subsequent PCR test identified *Plasmodium vivax*. The patient lived in a city close to where a malaria patient who had travelled to Pakistan had been diagnosed. A molecular comparison confirmed the same strain of *P. vivax* in both cases. Despite an exhaustive entomological investigation, not a single specimen of the anopheline vector was detected.

### Airport malaria

A number of cases which were classified as airport malaria have been reported. The first in 1984 [20] was a 76-year-old woman who developed fever with respiratory symptoms, which was initially diagnosed as pneumonia. Following the failure of the antibiotic treatment, the patient was hospitalized with a presumed bacteraemia. Finally, a microscopic analysis revealed *P. falciparum* gametocytes and numerous trophozoites. Despite treatment with quinine, the patient died a few days later due to respiratory complications. The disease had presented after a trip to visit a family member less than 6 km from Madrid airport.

In 2001, a 75-year-old woman who lived close to two international airports (4 and 18 km distance, respectively), was hospitalized with intermittent fever that had lasted a week although without apparent infection [21]. Microscopic examination of her blood showed the presence of intraerythrocytic rings. A rapid antigen test for *P. falciparum* and *P. vivax* was negative, and later tests showed *Plasmodium ovale*. A course of treatment with chloroquine and primaquine was initiated and the patient recovered.

### Congenital malaria

To date five cases of neonatal malaria have been identified in Spain. The first case, in 1999 [22], was a newborn whose mother was an expatriate that had lived in Equatorial Guinea until the 8 month of the pregnancy, and had been treated for malaria whilst there. In its 3rd week, the baby developed a fever without other associated symptoms, and the examination of a thick blood smear showed the presence of schizonts and gametocytes related to *P. falciparum*.

In 2007, an 8-week-old baby presented with fever and altered coagulation; the blood test identified *P. vivax*. The mother, an expatriate who had arrived in Spain from Senegal a year before, was asymptomatic.

A new case appeared in 2009 involving a 5-week-old baby suffering from anaemia and hepatosplenomegaly caused by *P. falciparum.* The mother, from Equatorial Guinea, had suffered from malaria during the pregnancy.

The fourth was reported in 2011. The mother was an expatriate who had come from Equatorial Guinea when she was in the 6th month of pregnancy. She was diagnosed there with malaria in the first third of the pregnancy. In consequence, the newborn was tested against malaria and *P. falciparum* was detected, although the baby was asymptomatic.

The fifth case appeared in 2014 [19]. The mother had returned a month before from a trip to Equatorial Guinea to visit her relatives. She gave birth prematurely at 35 weeks. A week later she presented with a fever which was diagnosed as *P. falciparum*. The newborn had been asymptomatic but blood tests revealed a malarial infection.

### Induced malaria

#### Transfusion-transmitted malaria

In 1971, there was a substantial outbreak of transfusion-transmitted malaria in Spain. 54 people were affected, 43 of whom had received full blood transfusions, while a further 11 had received plasmapheresis. It seems that the latter patients received blood cells from donors instead of receiving their own cells. The outbreak was caused by *P. vivax* and most of these cases were traced to a blood bank in Barcelona, which used workers from North and Central Africa as donors [23].

There have been three cases of transfusion-transmitted malaria since that outbreak. In 1987, there was a case of a 32-year-old woman who contracted *P. falciparum* after having received a transfusion after delivery [24]. She was hospitalized a week later with fever, and intraerythrocytic parasites were observed under microscopic analysis. The patient had received blood from two separate donors, who were duly investigated. In one of them, a traveler who had stayed in The Democratic Republic of Congo for 2 months, anti-malarial antibodies were detected.

A decade later a new case was identified, a 63-year-old woman who had had a transfusion. Three weeks afterwards the patient started to suffer fever without an apparent infection, and, 4 weeks later, a peripheral blood smear showed the presence of *P. falciparum*. Amongst the possible donors was an expatriate from a Central African country who had suffered from the disease a number of years before. This person lived in Spain but had travelled back to their country of origin a number of times without showing symptoms [25].

The last reported case was in 2002 when a 26-year-old woman [26] received a number of blood transfusions in addition to erythrocyte concentrate. The following month she presented with fever spikes and, later, a ruptured spleen. Blood cultures isolated a number of microorganisms but the antibiotic treatment administered failed to produce an improvement in her condition. Finally, a peripheral blood smear revealed *P. falciparum*; the delay in diagnosis, however—as 4 months had passed—did not allow the donor to be identified.

#### Post-transplant malaria

The first case of post-transplant Malaria in Spain was a 30-year-old man who had received a liver transplant [27] in 2005. Three weeks after the transplant, the patient presented with fever, shivers and hypotension. Molecular tests confirmed the presence of *P. vivax*. The donor had lived in Colombia up to 2004 and had suffered from malaria in 2001; however, he had been asymptomatic since the initial treatment. A further four patients who had received organs from the same donor remained asymptomatic, but in two of the four thick blood smear tests showed malaria parasites and they were treated with anti-malarials.

In 2005, there were two other cases of post-transplant malaria, both from the same Bolivian donor. Two women who had received transplants (kidney and heart respectively) developed fever a number of weeks after the procedure. Peripheral blood studies identified *P. vivax.*

In 2013, a 50-year-old man received a heart transplant [28] and 2 weeks later he developed fever and abdominal pain due to a splenic infarction. A peripheral blood analysis revealed intracellular parasites consistent with *P. falciparum*. The donor originally came from Mali and had arrived in Spain a year before, although it was not known if he had travelled subsequently. Another four patients received organs from the same donor, one of whom developed fever and the subsequent blood test was positive. A further patient—though asymptomatic—had a positive antigen test. Anti-malarial treatment was administered to both patients. The other two organ recipients, though not presenting symptoms and with negative blood smears, were given anti-malarial prophylaxis.

The last reported case was in 2014 [19] when a 52-year-old male who had received a kidney transplant presented a month afterwards with symptoms that were initially diagnosed as a urinary infection. A blood smear test showed the presence of *P. ovale*. The donor was Equatorial Guinean in origin and had travelled there recently. The other patients who received organs from the same donor were asymptomatic but nevertheless received anti-malarial treatment.

#### Parenteral transmission

Needle sharing among intravenous drug users has also been the origin of a number of malaria cases, principally *P. falciparum* [29]. In Spain, during the eighties, there were two outbreaks. The first—caused by *P. vivax*—affected five youths [30], and the second—caused by *P. falciparum*—infected two men [31]. In both outbreaks the affected patients had shared needles with people who had travelled to Equatorial Guinea.

#### Other nosocomial cases

In 1978, a case of nosocomial malaria was reported in Spain. The patient was a nurse that had been in contact with a malaria patient [32].

Subsequently, three further cases were reported with an epidemiological link to a hospital, but it was not possible to establish the mechanism of infection in any of those cases. A case involving a 70-year-old male was reported in 1998 [33]. He had undergone a surgical intervention but had not received a blood transfusion. The epidemiological investigation revealed that he had been in an emergency hospital room for a number of hours, close to another patient from Mauritania who had undergone an exsanguino transfusion and was later diagnosed with malaria.

In 2010 [34], a 68-year-old man suffered septic shock of unknown origin, and blood tests showed the presence of intraerythrocytic parasites, which were identified as *P. falciparum*. The patient, who had been hospitalized 2 weeks previously, had shared a room and nursing care with a malaria patient who had returned from a trip to Gambia. It was not possible to recover blood sample from the African patient in order to do molecular studies.

Finally, in August 2011 [35], a 4-year-old child presented with fever and liver involvement, and in the peripheral blood analysis *P. falciparum* was identified. The epidemiological investigation revealed that the child had been hospitalized 2 weeks earlier and that during his stay he had been close to a 6-year-old girl from Equatorial Guinea. Molecular typing was performed and the parasite populations in both patients matched.

Table 1 summarizes the episodes of non-imported malaria in Spain. Half of the cases were reported in the last 10 years. No introduced or post-transplant malaria cases were reported prior to 2005. In the last 10 years there have been no reported cases of transfusional malaria, airport malaria, or malaria due to parenteral infection. More than half of the reported cases were *P. falciparum*.

**Table 1:** Reported cases of non-imported malaria in Spain

Event classification	Year of diagnosis	*Plasmodium* identified	Cases	Relevant epidemiological data	
Introduced malaria	2010	*P. vivax*	1	The patient lived in an area with *Anopheles atroparvus*. No reported cases of imported malaria in previous years	[18]
	2014	*P. vivax*	1	The strains of both the patient and of a case with malaria that had travelled to Pakistan matched by molecular typing. No anopheline vector was detected in the area	[19]
Airport malaria	1984	*P. falciparum*	1	The patient visited a family member who lived less than 6 km from Madrid airport	[20]
	2001	*P. ovale*	1	The patient lived close to two international airports (4 and 18 km distance, respectively)	[21]
Congenital malaria	1999	*P. falciparum*	1	The mother was an expatriate and has been living in Equatorial Guinea until the 8 month of pregnancy	[22]
	2007	*P. vivax*	1	The mother, asymptomatic, have arrived to Spain a year before from Senegal	
	2009	*P. falciparum*	1	The mother originally came from Equatorial Guinea	
	2011	*P. falciparum*	1	The mother was an expatriate who had come from Equatorial Guinea when she was in the 6th month of pregnancy	
	2014	*P. falciparum*	1	The mother have travelled to Equatorial Guinea to visit her relatives	[19]
Induced malaria					
Transfusional	1971	*P. vivax*	54	43 cases received full blood transfusions +11 cases received plasmapheresis. Origen: a blood bank whose donors have different African origin: Algeria, Morocco and Equatorial Guinea	[23]
	1987	*P. falciparum*	1	The donor have travelled to Democratic Republic of Congo	[24]
	1997	*P. falciparum*	1	The possible donor was a Central African expatriate who have travelled several times to his country	[25]
	2002	*P. falciparum*	1	No known donor	[26]
Post-transplant	2005	*P. vivax*	3	The donor had lived in Colombia until the year before. One symptomatic recipient and two with positive smears	[27]
	2005	*P. vivax*	2	Bolivian donor. Both recipients had symptoms	
	2013	*P. falciparum*	3	The donor had arrived from Mali a year before. Two symptomatic recipients and one with a positive antigen test	[28]
	2014	*P. ovale*	1	The donor had travelled to Equatorial Guinea	[19]
Parenteral	1984	*P. vivax*	5	Needles shared with people who had travelled to Equatorial Guinea	[30]
	1986	*P. falciparum*	2	Needles shared with people who had travelled to Equatorial Guinea	[31]
Other nosocomial	1978	*P. falciparum*	1	A nurse was infected by a malarial patient	[32]
	1998	*P. falciparum*	1	The case shared room with and nursing care patient with malaria	[33]
	2010	*P. falciparum*	1	The case shared room and nursing care with a patient with malaria	[34]
	2011	*P. falciparum*	1	The case shared room with a patient with malaria. Molecular typing in both patients matched	[35]

Years 1964–2014

## Discussion

In non-endemic countries malaria is generally an imported disease that must be taken into account in differential diagnoses when travellers become ill after returning from endemic areas. When a history of such travel is not present the diagnosis of malaria is highly improbable but should not be excluded. The data from Spain demonstrates cases infected locally account for around 0.8% of the total cases since the disease was declared eradicated. It is not always easy to determine the mode of transmission and it is crucial that an exhaustive history of possible hospitalizations or recent transfusions is recorded. In non-endemic countries, control measures for the selection of donors are in place both for blood and organs. However, the transmission of malaria by these routes cannot be discarded. Sometimes, the information gathered during the interview prior to donating blood is not correct, and in other cases the malarial infection, especially that caused by *Plasmodium malariae* can persist in the host for years in a form which is subclinical [36] with parasite levels low enough to escape detection by peripheral blood analysis or through antigen detection, as these tests are not sufficiently sensitive.

The clinical history must also include data regarding the location of the patient’s home and work, and their possible proximity to an international airport. To avoid cases of airport malaria it is essential to follow the WHO International Health Regulations [37], and to implement vector control programmes, both in aircraft coming from endemic areas and in the area which surrounds the airport (400 metres).

When the previously mentioned scenarios have been eliminated, it is necessary to consider the possibility of local transmission in areas where an appropriate vector exists, and where there are travellers who could act as potential carriers of gametocytes. In Spain, according to a report prepared by the European Centre for Disease Prevention and Control, the reintroduction of malaria is unlikely. This is because the principal potential vector in Spain, *Anopheles atroparvus*—although widely distributed—is competent to transmit Asian strains of *P. vivax,* but is impervious to African strains of *P. falciparum*, which is responsible for the majority of imported cases in Spain [38–40]. That said, the appearance of isolated cases remains a possibility which makes epidemiological and entomological vigilance necessary, particularly in the current context of climate change and globalization.

With respect to cases of congenital malaria, it is essential to take into account that the interval between maternal exposure and the effect on the newborn can be of great duration, and that the mother may even present as asymptomatic.

## Conclusions

Non-imported cases of malaria in non-endemic countries presuppose an important duty on the part of the health services, especially as their rarity is usually associated with a delayed diagnosis, which—taken alongside the elevated mortality of *P. falciparum*—can have serious consequences for the patient.

## References

[1] WHO. World Malaria Report. Geneva: World Health Organization; 2016. http://apps.who.int/iris/bitstream/10665/252038/1/9789241511711-eng.pdf?ua=1.

[2] Isaacson M (1989). Airport malaria: a review. Bull WHO.

[3] Gallien S, Taieb F, Hamane S, De Castro N, Molina JM (2013). Autochthonous falciparum malaria possibly transmitted by luggage-carried vector in Paris, France, February 2013. Euro Surveill.

[4] WHO. Update on malaria terminology. http://www.who.int/malaria/mpac/mpac-sept2015-terminology-annex2.pdf?ua=1.

[5] Uneke CJ (2007). Congenital *Plasmodium falciparum* malaria in sub-Saharan Africa: a rarity or frequent occurrence?. Parasitol Res.

[6] Lesko CR, Arguin PM, Newman RD (2007). Congenital malaria in the United States: a review of cases from 1966 to 2005. Arch Pediatr Adolesc Med.

[7] Kitchen AD, Chiodini PL (2006). Malaria and blood transfusion. Vox Sang.

[8] Chiche L, Lesage A, Duhamel C, Salame E, Malet M, Samba D (2003). Posttransplant malaria: first case of transmission of *Plasmodium falciparum* from a white multiorgan donor to four recipients. Transplantation.

[9] Martín-Dávila P, Fortún J, López-Vélez R, Norman F, Montes de Oca M, Zamarrón P (2008). Transmission of tropical and geographically restricted infections during solid-organ transplantation. Clin Microbiol Rev.

[10] Tarantola AP, Rachline AC, Konto C, Houzé S, Lariven S, Fichelle A (2004). Occupational malaria following needlestick injury. Emerg Infect Dis.

[11] González L, Ochoa J, Franco L, Arroyave M, Restrepo E, Blair S (2005). Nosocomial *Plasmodium falciparum* infections confirmed by molecular typing in Medellín, Colombia. Malar J..

[12] Chen KT, Chen CJ, Chang PY, Morse DL (1999). A nosocomial outbreak of malaria associated with contaminated catheters and contrast medium of a computed tomographic scanner. Infect Control Hosp Epidemiol.

[13] Piro S, Sammud M, Badi S, Al Ssabi L (2001). Hospital-acquired malaria transmitted by contaminated gloves. J Hosp Infect.

[14] Rico-Avello C. Historia del paludismo en España. Rev San Hig Pública. 1947. Nº 5;483–525.

[15] Pletsch D (1965). Informe sobre una misión efectuada en España en septiembre–noviembre de 1963 destinada a la certificación de la erradicación del paludismo. Rev San Hig Pública..

[16] Velasco E, Díaz O (2014). Cincuenta años sin paludismo. Bol Epidemiol Semanal..

[17] del Clavero G (1961). La erradicación del paludismo en España. Rev San Hig Pública..

[18] Santa-Olalla Peralta P, Vazquez-Torres MC, Latorre-Fandós E, Mairal-Claver P, Cortina-Solano P, Puy-Azón A (2010). The first autochthonous malaria case due to *Plasmodium vivax* since eradication, Spain, October 2010. Euro Surveill.

[19] Resultados de la vigilancia epidemiológica de las enfermedades transmisibles. Informe anual. Año 2014. Instituto de Salud Carlos III.

[20] Alós JI, Cercenado E, Rodríguez-Créixems M, Erice A, Bouza E (1985). Suspected airport malaria in Spain. Eur J Clin Microbiol..

[21] Cuadros J, Calvente MJ, Benito A, Arévalo J, Calero MA, Segura J (2002). *Plasmodium ovale* malaria acquired in central Spain. Emerg Infect Dis..

[22] Romero Urbano JM, Vázquez López R, Jurado Ortiz A, Martínez Valverde A (2000). Un caso de paludismo neonatal en España. An Esp Pediatr.

[23] Bruce-Chwatt LJ (1974). Transfusion malaria. Bull World Health Organ.

[24] Notificación de un caso de paludismo por transfusión. Boletín Epidemiológico Semanal. Ministerio de Sanidad y Consumo 1987; nº 1773. Sem.7.

[25] Pizarro Portillo A, García Polo I, Fernández Dorado MT, Delgado Meliá T (1998). Transfusion induced malaria. Rev Clin Esp.

[26] Tejero R, Gallego C, Lacasa MJ, Muñoz J (2003). Fiebre nosocomial de origen incierto en una paciente politraumatizada. Enferm Infecc Microbiol Clin.

[27] Rodriguez M, Tome S, Vizcaino L, Fernandez-Castroagudin J, Otero-Anton E, Molina E (2007). Malaria infection through multiorgan donation: an update from Spain. Liver Transpl.

[28] Sabé N, González-Costello J, Oriol I, Sánchez-Salado JC, Ortega S, Oliver E (2014). Donor-transmitted malaria after heart transplant managed successfully with artesunate. Transpl Infect Dis..

[29] Bick RL, Anhalt JE (1971). Malaria transmission among narcotic addicts. A report of ten cases and review of the literature. Calif Med..

[30] González García J, Arnalich F, Peña JM, Garcia-Alegria JJ, Garcia Fernandez F, Jimenez Herraez C (1986). An outbreak of *Plasmodium vivax* malaria among heroin users in Spain. Trans R Soc Trop Med Hyg.

[31] Vigilancia del paludismo. Segundo semestre de 1986. Boletín epidemiológico semanal. Ministerio de Sanidad y Consumo. 1987; nº 1772.

[32] Investigación entomológica sobre receptividad al paludismo. Boletín Epidemiológico Semanal. Ministerio de Sanidad y Consumo. 1978; nº1336. Sem.27.

[33] Rotaeche Montalvo V. Paludismo inducido en España. 1971–2000. Boletín Epidemiol Semanal. 2001;9: 137-8.

[34] Resultados de la vigilancia epidemiológica de las enfermedades transmisibles. Informe anual. Año 2010. Instituto de Salud Carlos III.

[35] Resultados de la vigilancia epidemiológica de las enfermedades transmisibles. Informe anual. Año 2011. Instituto de Salud Carlos III.

[36] Brouwer EE, van Hellemond JJ, van Genderen PJ, Slot E, van Lieshout L, Visser LG (2013). A case report of transfusion-transmitted *Plasmodium malariae* from an asymptomatic non-immune traveller. Malar J..

[37] WHO. International Health Regulations. 2005. http://www.who.int/ihr/publications/9789241596664/en/.

[38] ECDC. Consultation on *Plasmodium vivax* transmission risk in Europe. Stockholm, 17–18 January 2012. Meeting report. http://ecdc.europa.eu/en/publications/_layouts/forms/Publication_DispForm.aspx?ID=596&List=4f55ad51-4aed-4d32-b960-af70113dbb90.

[39] Delacour S, Melero-Alcibar R, Aranda C, Cortés M, Eritja R, Escosa R et al. Detailed maps of the geographical distribution of the mosquitoes of Spain based on a literature review. Part II: Genus *Anopheles*. In: The 5th European Mosquito Control Association Workshop. Turin; 2009.

[40] de Zulueta J, Ramsdale CD, Coluzzi M (1975). Receptivity to malaria in Europe. Bull World Health Organ.

